# County-level racial disparities in prostate cancer–specific mortality from 2005 to 2020

**DOI:** 10.1093/jncics/pkae109

**Published:** 2024-11-04

**Authors:** Samuel L Washington III, Mary Fakunle, Lufan Wang, Avery E Braun, Michael Leapman, Janet E Cowan, Matthew R Cooperberg

**Affiliations:** Department of Urology, Helen Diller Family Comprehensive Cancer Center, University of California, San Francisco, CA 94143, United States; Department of Epidemiology & Biostatistics, University of California, San Francisco, San Francisco, CA 94143, United States; Department of Urology, Helen Diller Family Comprehensive Cancer Center, University of California, San Francisco, CA 94143, United States; Department of Urology, Helen Diller Family Comprehensive Cancer Center, University of California, San Francisco, CA 94143, United States; Department of Urology, Helen Diller Family Comprehensive Cancer Center, University of California, San Francisco, CA 94143, United States; Department of Urology, Yale University, New Haven, CT 06510, United States; Department of Urology, Helen Diller Family Comprehensive Cancer Center, University of California, San Francisco, CA 94143, United States; Department of Urology, Helen Diller Family Comprehensive Cancer Center, University of California, San Francisco, CA 94143, United States; Department of Epidemiology & Biostatistics, University of California, San Francisco, San Francisco, CA 94143, United States

## Abstract

**Background:**

Local conditions where people live continue to influence prostate cancer outcomes. By examining local characteristics associated with trends in Black-White differences in prostate cancer–specific mortality over time, we aim to identify factors driving county-level prostate cancer–specific mortality disparities over a 15-year period.

**Methods:**

We linked county-level data (Area Health Resource File) with clinicodemographic data of men with prostate cancer (Surveillance, Epidemiology, and End Results registry) from 2005 to 2020. Generalized linear mixed models evaluated associations between race and county-level age-standardized prostate cancer–specific mortality, adjusting for age; year of death; rurality; county-level education; income; uninsured rates; and densities of urologists, radiologists, primary care practitioners, and hospital beds.

**Results:**

In 1085 counties, 185 390 patients were identified of which 15.8% were non-Hispanic Black. Racial disparities in prostate cancer–specific mortality narrowed from 2005 to 2020 (25.4 per 100 000 to 19.2 per 100 000 overall, 57.9 per 100 000 to 38 per 100 000 for non-Hispanic Black patients, and 23.4 per 100 000 to 18.3 per 100 000 for non-Hispanic White patients). For non-Hispanic Black and non-Hispanic White patients, county prostate cancer–specific mortality changes varied greatly (-65% to +77% and -61% to +112%, respectively). From 2016 to 2020, non-Hispanic Black patients harbored greater prostate cancer–specific mortality risk (relative risk = 2.09, 95% confidence interval [CI] = 2.01 to 2.18); higher radiation oncologist density was associated with lower mortality risk (relative risk = 0.93, 95% CI = 0.89 to 0.98), while other practitioner densities were not.

**Conclusion:**

Although overall rates improved, specific counties experienced worsening race-based disparities over time. Identifying locations of highest (and lowest) mortality disparities remains critical to development of location-specific solutions to racial disparities in prostate cancer outcomes.

## Introduction

Prostate cancer remains the second most common cause of cancer deaths nationally;^1^ local conditions related to where people live, learn, work, and play (ie, social determinants of health) continue to influence cancer outcomes.^1^^,^^2^ Overall trends in prostate cancer–specific mortality continue to demonstrate disproportionate burdens of disease across racial groups as Black patients harbor a greater than 2-fold risk compared with White counterparts nationally.^1^^,^^3^ Comparing prostate cancer–specific mortality rates from 1990 to 1994 and 2005 to 2009, Black patients experienced up to 3 times greater mortality rates than White counterparts across 37 of the largest US cities. The observed level of disparity has been associated with the extent of residential segregation within cities, and excess deaths due to these disparities vary over time at the local level.^4^

Although population-level analyses have focused awareness of the existence of racial disparities in prostate cancer burden and improvement nationally, these effects are not uniformly distributed.^1^^,^^5^ Our understandings of small area–level drivers of worsening racial disparities remain limited. Regional- and race-based differences in medical resources similarly impact timely access to screening, treatment, care delivery, and ultimately cancer outcomes for Black and White patients.^6^ Living in neighborhoods with lower education and/or income is associated with worse prostate cancer outcomes compared with residing in areas of greater socioeconomic status.^6^^,^^7^ These studies highlight the growing need to identify where disparities are worsening and what region-specific factors contribute to their persistence over time. By examining contemporary measures of regional characteristics and medical resources associated with Black-White differences in prostate cancer–specific mortality over time, we aimed to examine county-level changes in racial disparities in prostate cancer–specific mortality over a 15-year period to identify factors associated with worsening local prostate cancer age-standardized mortality rates. We aim to leverage these findings as the groundwork for area-specific interventions specific to regions with worsening racial disparities.

## Methods

In this study, we linked county-level measures of health-care resources within the Area Health Resource File (AHRF) and clinicodemographic data on patients diagnosed with prostate cancer in the Surveillance, Epidemiology, and End Results (SEER) registry. Patient-level characteristics and county-level prostate cancer–specific mortality rates were collected from the SEER registry for patients diagnosed with prostate cancer within 3 time periods: T1 (2005-2010), T2 (2011-2015), and T3 (2016-2020). The SEER data available was previously provided for the following time periods: 2005-2007, 2008-2010, 2011-2015, and 2016-2020, which were collapsed into 3 uniform time periods to control for time-based changes in unmeasured confounders present within these datasets. The AHRF is produced by the US Department of Health and Human Services and provides data on health-care practitioners by specialty, health facilities in terms of number of beds within each facility, and population demographics, all at the county and state levels. From AHRF, we collected county-level data, including household income, insurance status, and practitioner density (urologist, radiation oncologist, primary care practitioner per 100 000). AHRF measures were linked to patient-level data on county of residence within the SEER cohort by matching based on the combined Federal Information Processing System county codes, as we have published previously.^8^

The primary outcome was county-level age-standardized mortality rates for prostate cancer. Patient-level clinicodemographic data were abstracted from SEER including race and ethnicity (defined as non-Hispanic Black, non-Hispanic White, and Hispanic) and age (categorized as aged younger than 50 years, 50-64 years, and 65 years and older). Generalized linear mixed models with negative binomial distribution evaluated associations between race and contemporary age-standardized mortality rates (2016-2020). Fixed effects in the models included age (younger than 50 years, 50-64 years, and 65 years and older); year of death; rurality (defined using 3-level rural-urban continuum) and county-level proportions of residents with a 4-year college degree; median household income; percentage of uninsured patients aged younger than 65 years (quartiles); and densities of urologists, radiologists, primary care practitioners, and hospital beds per 100 000 persons. Additional 2-level hierarchical modeling with counties nested within SEER registries as random effects assessed factors associated with age-standardized mortality rates over time (2005-2020) using rate ratios and 95% confidence intervals (CIs).

We calculated age-adjusted prostate cancer–specific mortality rates from T1, T2, and T3 for non-Hispanic White and non-Hispanic Black patients. Comparing time points T1 and T3, we extrapolated age-adjusted prostate cancer–specific mortality rates and calculated percent changes in prostate cancer–specific mortality for non-Hispanic White and non-Hispanic Black patients. We examined changes in mortality rate ratios for non-Hispanic Black to non-Hispanic White patients within each SEER registry over the last 10 years (2011-2020) and restricted to the most recent time period (T3, 2016-2020). Generalized linear regression models, again using a negative binomial distribution of the number of deaths, explored the associations between age-adjusted prostate cancer–specific mortality and covariates within the most contemporary time period (T3, 2016-2020) (model 1). Additional models explored the associations between the variables and age-adjusted prostate cancer–specific mortality over the 3 time points (model 2). In model 2, counties were further nested within SEER registries in a 2-level hierarchical structure.

## Results

In total, 185 390 patients with prostate cancer as the reported cause of death were identified in 1085 counties. Based on those included, 15.8% were Non-Hispanic Black and 8.9% Hispanic. The most prevalent age group was patients aged 65 years or older (88.8%), which accounted for 90.8% of non-Hispanic White patients and 81.3% of Non-Hispanic Black patients. Most counties were categorized as metropolitan (41%) or urban (44%; Table 1). One-half (52%) reported 15 000-30 000 residents per 100 000 persons with a college education, and most (65%) reported a median annual household income of less than $50 000. Most counties had no urologist (66%), 0-1 radiation oncologists (79%), and less than 50 primary care practitioners (57%).

**Table 1.** Patient and county-level characteristics of men diagnosed with prostate cancer from 2005 to 2020.

Characteristic	No. (%)
Patient characteristics	
Race	
Non-Hispanic White	139 548 (75.3)
Non-Hispanic Black	29 345 (15.8)
Hispanic ethnicity	16 497 (8.9)
Age group (in years)	
<50	14 (0.01)
50-64	12 685 (6.8)
65+	16 4596 (88.8)
Unknown	8095 (4.4)
County characteristics (n = number of counties)	
Rural-Urban continuum	
Metropolitan areas	440 (41)
Urban areas	476 (44)
Rural areas	169 (16)
Completion of 4 + yr college education (per 100 000)	
<15 000	367 (34)
15 000-30 000	565 (52)
30 000-45 000	127 (12)
≥45 000	26 (2)
Median household income	
<$50 000	701 (65)
$50 000-75 000	339 (31)
≥$75 000	45 (4)
%<65 years old men without insurance	
1st quartile	274 (25)
2nd quartile	272 (25)
3rd quartile	275 (25)
4th quartile	264 (24)
# Urologists per 100K	
<1	714 (66)
1-4	240 (22)
>4	131 (12)
# Radiation Oncologists per 100K	
0	797 (73)
<1	69 (6)
1-2	112 (10)
2-3	63 (6)
>3	44 (4)
# Primary Care Practitioners per 100K	
<50	621 (57)
≥50	464 (43)
# Hospital beds per 100K	
0	239 (22)
<200	347 (32)
200-400	306 (28)

**Figure 1.**
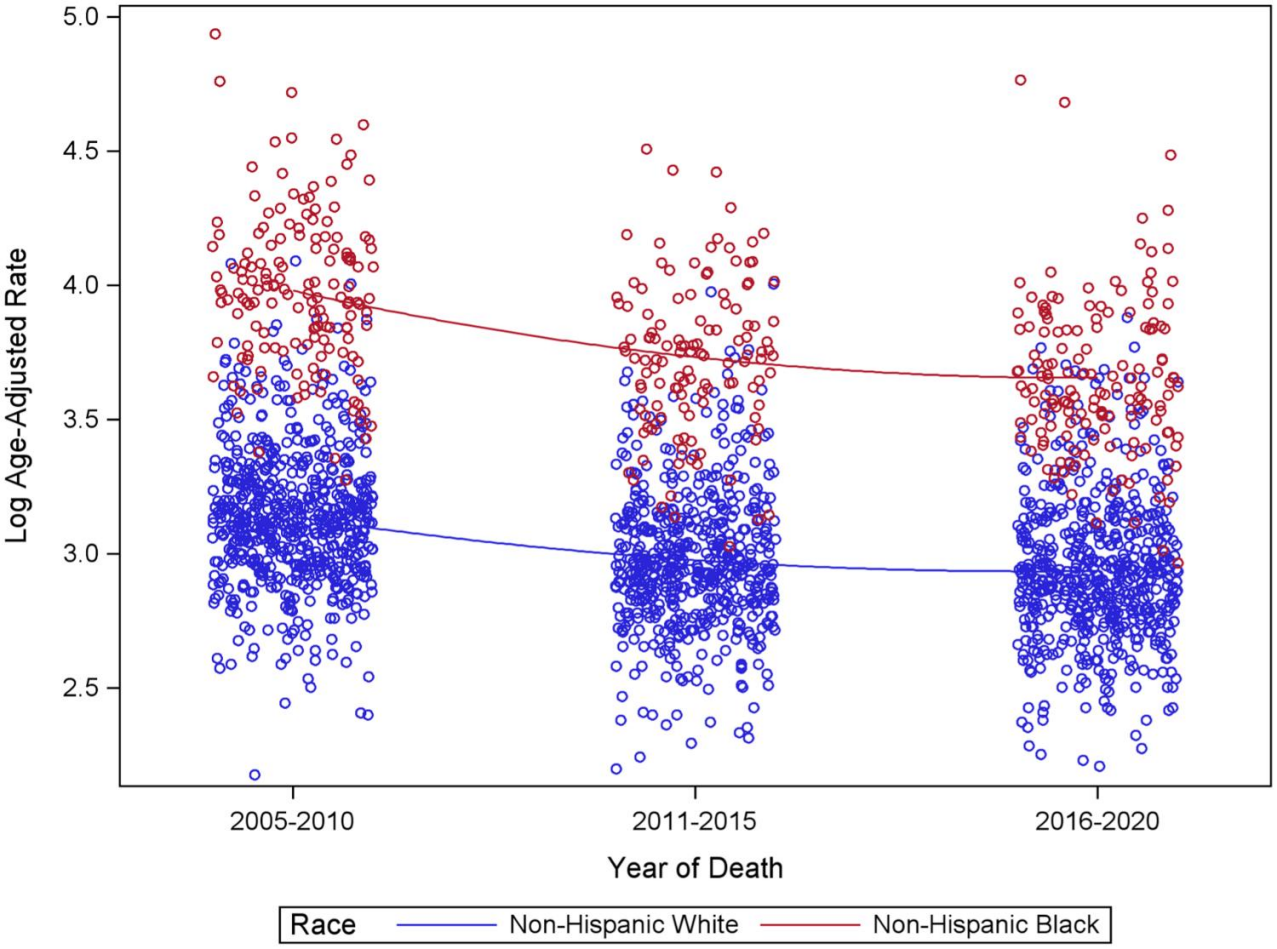
Trend of age-standardized county-level rates of prostate cancer–specific mortality from 2005-2010 (T1) to 2016-2020 (T3) of the 2 race groups (rates per 100 000 population). Each point represents an individual county rate during the specified time interval.

**Figure 2.**
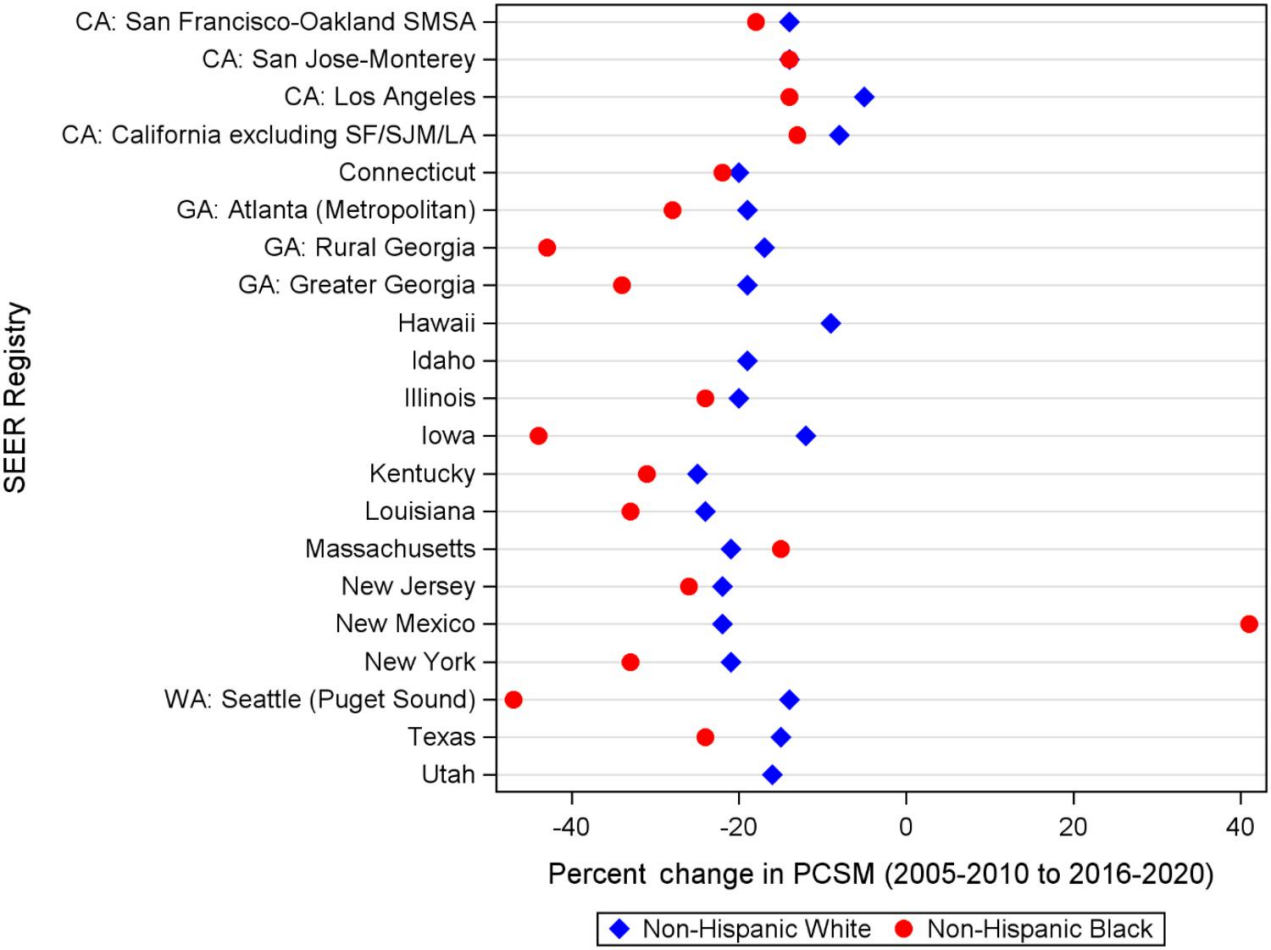
Percent change of prostate cancer-specific mortality between 2005-2010 (T1) and 2016-2020 (T3) for Non-Hispanic White and non-Hispanic Black men, by Surveillance, Epidemiology, and End Results registry. Percent change is calculated by 100*(PCSM at T3—PCSM at T1)/PCSM at T1. LA = Los Angeles; PCSM = prostate cancer–specific mortality; SEER = Surveillance, Epidemiology, and End Results; SF = San Francisco; SJM = San Jose/Monterey; SMSA = San Francisco-Oakland SMSA (Standard Metropolitan Statistical Area).

**Figure 3.**
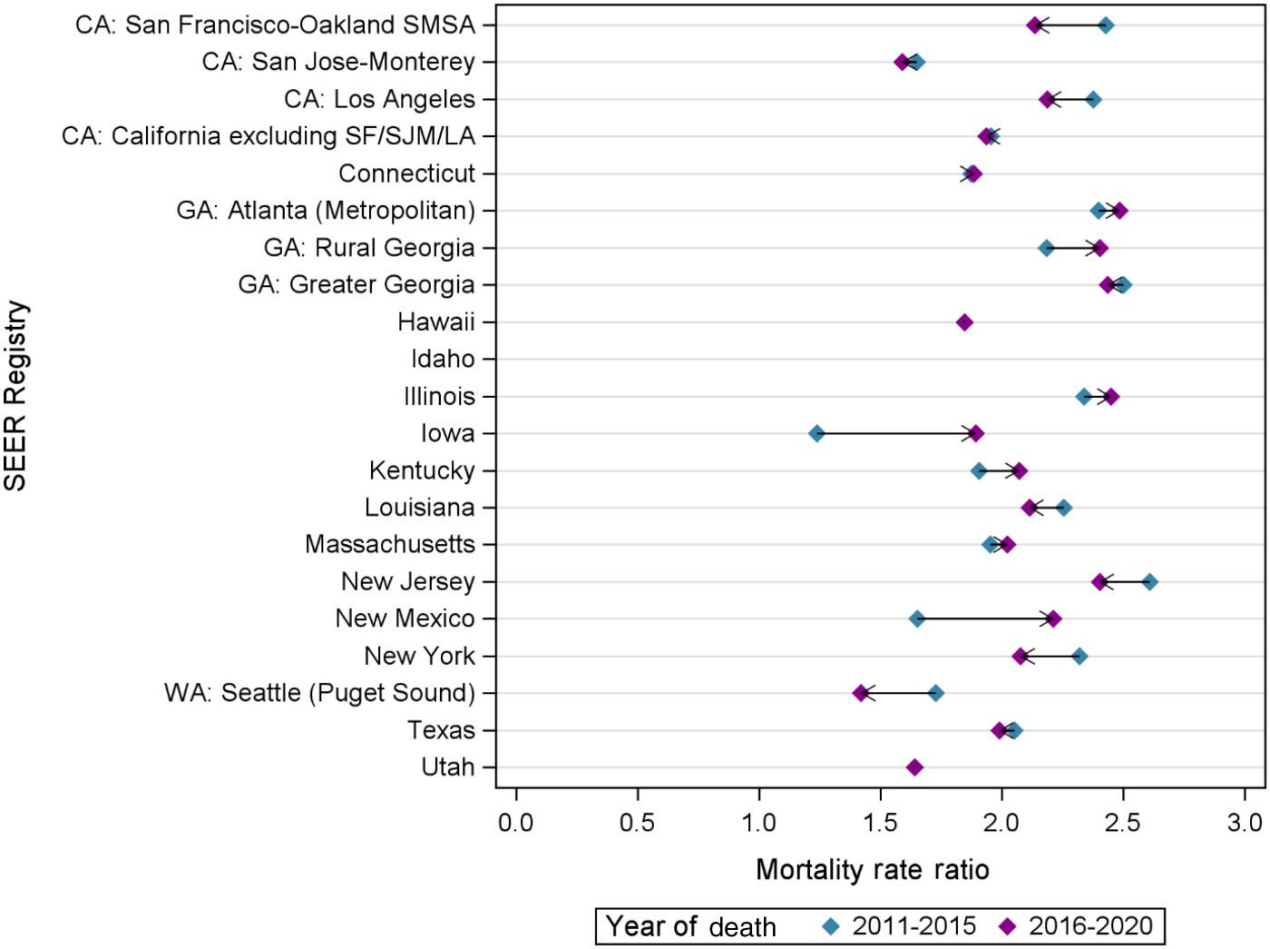
Prostate cancer-specific mortality rate ratios of Non-Hispanic Black men vs Non-Hispanic White men over time. **Arrows** shows direction of change from 2011-2015 to 2016-2020. LA = Los Angeles; SEER = Surveillance, Epidemiology, and End Results; SF = San Francisco; SJM = San Jose/Monterey; SMSA = San Francisco-Oakland SMSA (Standard Metropolitan Statistical Area).

From 2005 to 2020, county-level age-standardized mortality rates for on-Hispanic White patients (from 23.0 per 100 000 to 18.3 per 100 000) and Non-Hispanic Black patients (from 54.1 per 100 000 to 37.6 per 100 000) decreased over time. Non-Hispanic Black patients experienced a greater decrease in age-standardized mortality rates compared with Non-Hispanic White patients (30.5% change compared with 20.4% for non-Hispanic White patients); yet age-standardized mortality rates for Non-Hispanic Black patients remained higher compared with Non-Hispanic White patients across all time periods (Figure 1). We then examined the county-level percent change in prostate cancer–specific mortality between the first (2005-2010) and last (2016-2020) time periods for Non-Hispanic White and Non-Hispanic Black patients, grouped by SEER registry (Figure 2). Percent changes in prostate cancer–specific mortality for Non-Hispanic Black patients varied greatly across counties, ranging from a 65% decrease in Bulloch County, Georgia, to at least a 77% increase in Peoria County, Illinois (Table S1). For Non-Hispanic White patients, percent changes in prostate cancer–specific mortality ranged from a 61% decrease in Muscatine County, Iowa, to at least a 112% increase in Mason County, Illinois. Across SEER registries, disparities in mortality worsened in Connecticut, Massachusetts, Georgia (Atlanta, rural Georgia), Illinois, New Mexico, Kentucky, and Iowa (Figure 3).

When examining county-level factors associated with age-standardized mortality rates over the entire study period (2005-2020), risk decreased overall over time (2016-2020: rate ratio = 0.8, 95% CI = 0.79 to 0.82; 2011-2015: rate ratio = 0.85, 95% CI = 0.83 to 0.86; Table S2, model 1). However, Non-Hispanic Black patients faced greater risk compared with Non-Hispanic White counterparts (rate ratio = 2.37, 95% CI = 2.21 to 2.53) even after adjustments. Younger age (50-64 years: rate ratio = 0.09, 95% CI = 0.09 to 0.09; younger than 50 years: rate ratio = 0.09, 95% CI = 0.09 to 0.09) and greater household income (≥$75 000: rate ratio. = 0.90, 95% CI = 0.87 to 0.94) were associated with lower risk of mortality. Higher primary care physician (PCP) density (≥50 per 100,000: rate ratio = 1.05, 95% CI = 1.02 to 1.08) and living in rural areas (rate ratio = 1.13, 95% CI = 1.04 to 1.24) were associated with greater risk of prostate cancer–specific mortality. For non-Hispanic White patients, greater PCP density was associated with increased mortality risk (rate ratio = 1.05, 95% CI = 1.02 to 1.08), whereas PCP density was not associated with mortality for non-Hispanic Black patients. Specialist density was not associated with mortality risk. After nesting county within SEER region, mortality risk for non-Hispanic Black patients remained although slightly lower (rate ratio = 2.15, 95% CI = 2.1 to 2.2) with unchanged overall risk over time (Model 2, Table S2). Risk associated with younger age, greater household income, PCP density, and living in rural areas remained largely unchanged. Greater PCP density remained associated with greater risk for non-Hispanic White patients but not non-Hispanic Black patients.

**Figure 4.**
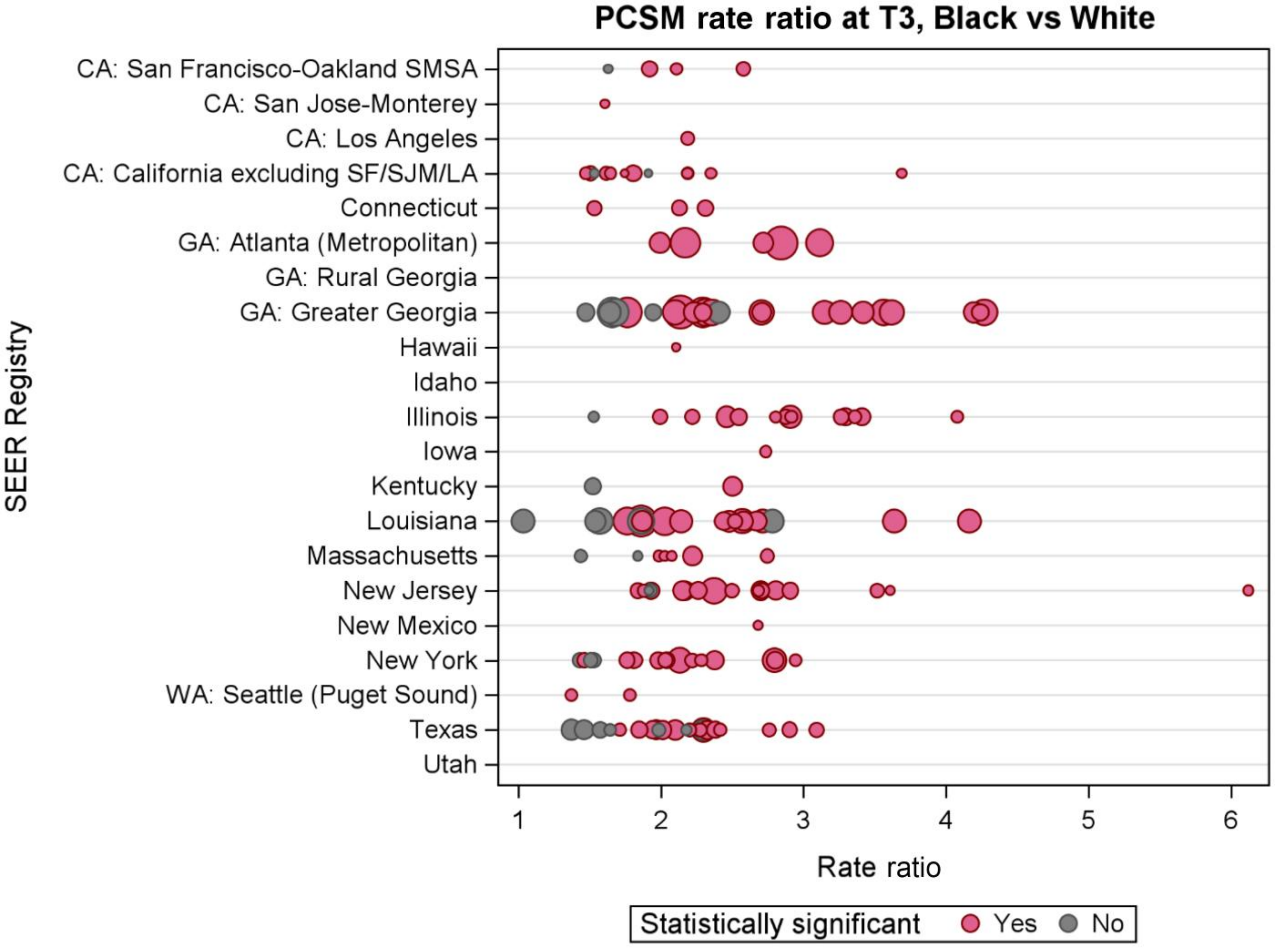
Mortality rate ratio comparing Non-Hispanic Black men vs Non-Hispanic White men (referent) at T3 in counties with available data. **Circle size** represents percent of Black men in each county. LA = Los Angeles; PCSM = prostate cancer–specific mortality; SEER = Surveillance, Epidemiology, and End Results; SF = San Francisco; SJM = San Jose/Monterey; SMSA = San Francisco-Oakland SMSA (Standard Metropolitan Statistical Area).

Comparing non-Hispanic Black with non-Hispanic White patients, the mortality rate ratio for non-Hispanic Black patients remained elevated across multiple counties within each SEER registry from 2016 to 2020 (Figure 4). Within this time interval (T3, 2016-2020), prostate cancer–specific mortality risk for non-Hispanic Black patients decreased slightly but remained greater than risk for non-Hispanic White patients (rate ratio = 2.09, 95% CI = 2.01 to 2.18; Table S3). Younger age, greater household income (≥$75 000), greater density of college graduates (≥45 per 100 000: rate ratio = 0.89, 95% CI = 0.81 to 0.98; 30 000-45 000: rate ratio = 0.91, 95% CI = 0.84 to 0.98) and higher county-level incomes (≥$75 000: rate ratio = 0.91, 95% CI = 0.86 to 0.96) remained associated with lower mortality risk. Radiation oncologist density of 1-2 per 100 000 persons was associated with lower risk of mortality (rate ratio = 0.93, 95% CI = 0.89 to 0.98), whereas PCP density was not.

## Discussion

In this study, we analyze county-level trends in racial disparities in prostate cancer–specific mortality from 2005-2020 using the SEER and AHRF databases. Although overall disparities in prostate cancer–specific mortality decreased, marked heterogeneity in changes were seen over time, and racial disparities worsened in specific regions of the United States (Connecticut, Georgia, Illinois, New Mexico, Kentucky, and Iowa). Higher regional educational ascertainment and radiation oncologist density was associated with lower mortality risk, but PCP density was not, within the most recent time period only.

We noted age-standardized mortality rates decreased from 2005 to 2020 nationally, although the improvement was not uniformly distributed, and prostate cancer–specific mortality rates for Black patients remained higher than those of White patients. Prostate cancer care patterns are well-known to feature racial and geographic disparities, but our understanding is limited in terms of localizing and examining worsening disparities in specific counties.^8-10^ National trends have highlighted the larger issue, but a focus on variations within smaller geographic areas will be necessary to identify causes and local solutions. Prior work has explored regional changes in prostate cancer disparities over time.^4^^,^^11^ One study noted worsening incidence rates of distant disease from 2007 to 2013 with incidence rates for White patients decreasing earlier than those of Black patients.^11^ In addition, incidence rates increased with worsening poverty levels and were greatest in the Northeast region. County-level time series analyses of cause-specific mortality by race and ethnicity from 2000 to 2019 observed that regions with higher absolute inequality also had higher malignancy-related mortality rates because of widespread and variable county-level disparities.^12^ Our findings take this observation further by identifying specific counties of worsening prostate cancer–specific mortality disparities and identifying local drivers. These findings justify further investigation into the structural drivers of racial disparities in mortality.

Our study builds on prior efforts by identifying counties with worsening racial disparities in prostate cancer–specific mortality and revealing local drivers such as rurality of residence, regional educational ascertainment, and practitioner density that could not be explored previously because of limited geographic granularity and availability of these measurements within a single dataset. Using the National Center for Health Statistics to examine disparity trends between Black and White individuals from 2000 to 2020, one study showed decreasing but persistent disparities in age-adjusted prostate cancer mortality rate ratio for prostate cancer (from 2.49, 95% CI = 2.41 to 2.56, in 2000 to 2.08, 95% CI = 2.02 to 2.14 in 2020) nationally.^13^ Our findings complement these observations well by also demonstrating an overall decrease in age-standardized mortality rates nationally with persistent county-level disparities in mortality over a similar study period. Taken further, our findings shift the focus from national trends to smaller geographic areas to understand drivers that influence outcomes. Prior work on city-level Black-White age-adjusted prostate cancer–specific mortality rates from 1990-1994 and 2005-2009 showed statistically significant increases in disparities with notable variation in 41 major US cities.^4^ This study used several factors specific to the local social and economic environment including race-specific indices of isolation, overall and race-specific median household incomes, percentages of those with a high school education and those without health insurance, and population size. The authors posited that these local measures could inform rapid city-level policy change to impact larger populations at risk. Our study represents a contemporary update for these studies with more comprehensive measures of medical resources, socioeconomics, and broader geographic inclusion to understand local sociodemographic and medical resource–related drivers of racial disparities.

This analysis is subject to several limitations. First, our findings are subject to bias because of factors unmeasured in retrospective studies. The combination of SEER and AHRF supplements variables missing from the other and allows for a more granular assessment of geographic drivers that incorporates SEER region- and county-level data. The combination of these datasets for more comprehensive analyses of patient and county-level factors associated with worse prostate cancer–specific mortality but cannot be used to draw conclusions about individuals within the study cohort. Second, the AHRF data provides measures of health-care resources but does not quantify access, utilization, or referral patterns within each county. This analysis operates under the assumption that care was provided locally and highlights the need for more future efforts to better characterize local patterns of care. Third, each dataset contains a dynamic cohort that may not account for population density changes or measures of the degree of racial segregation over time. We used age-standardized mortality rates to standardize comparisons across the numerous counties included regardless of the population size. Lastly, using data over a 15-year period allows for longitudinal trends in racial disparities within counties but does not account for local and regional policy changes that may also impact clinical outcomes over time. This limitation further highlights the need for detailed studies focusing on local drivers of health outcomes as well as city-, county-, and state-level policy changes that may have also contributed to their perpetuation.

Our study also has several strengths. Leveraging more recent data from SEER and the AHRF provides an updated assessment of racial disparities in prostate cancer–specific mortality, identifies specific counties with worsening disparities, and elucidates how local drivers associated with worse mortality change over time. Although the combination of these datasets provides a more comprehensive context for the observations, each dataset is currently limited in granularity to identify specific county-level changes or drivers of changes. These observations of geographic variations in disparities represent a necessary pre-implementation step to allow future works to design region-specific interventions. This study spans a 15-year interval of time, which facilitates analysis of how factors associated with worsening disparities may change over time. Although more recent publications have used a similar approach, these studies have not provided findings specific to prostate cancer and often have limited data on modifiable health-care metrics such as specialist density. These novel findings support future work to localize interventions to counties of worsening disparities and highlight counties with improving outcomes as possible locales to understand mitigation strategies.

Specific counties are subject to worsening racial disparities in prostate cancer–specific mortality with local drivers changing over time. Examining local disparities in age-standardized mortality rates identifies local drivers of prostate cancer–specific mortality disparities not captured with national or large regional observations. Identifying locations of highest (and lowest) mortality disparities remains critical to location-specific solutions to racial disparities in prostate cancer outcomes.

## Supplementary Material

pkae109_Supplementary_Data

## Data Availability

The datasets were derived from sources in the public domain: https://seer.cancer.gov. Any data analysis scripts that generated the results are available upon request.
