# Supplementary material for: County-level racial disparities in prostate cancer–specific mortality from 2005 to 2020
Source: JNCI Cancer Spectr. 2024 Nov 4;8(6):pkae109. doi: 10.1093/jncics/pkae109 (PMC11631307; doi:10.1093/jncics/pkae109)
Supplement: pkae109_Supplementary_Data [file pkae109_supplementary_data.zip › Supplemental Table 1 county data.docx]

**Supplementary Materials**

**Supplementary Table 1.**

**State-county level percent changes in prostate cancer specific mortality in non-Hispanic Black and White men over time.**

| SEER regions | State-county | Non-Hispanic White | Non-Hispanic Black |
| --- | --- | --- | --- |
| GA: Rural Georgia | Washington County | . | -20 |
| Iowa | Black Hawk County | 24 | . |
| Iowa | Boone County | -2 | . |
| Iowa | Bremer County | -43 | . |
| Iowa | Calhoun County | 5 | . |
| Iowa | Cass County | 89 | . |
| Iowa | Cedar County | 34 | . |
| Iowa | Cerro Gordo County | -15 | . |
| Iowa | Clarke County | -11 | . |
| Iowa | Clayton County | -15 | . |
| Iowa | Clinton County | -39 | . |
| Iowa | Crawford County | -10 | . |
| Iowa | Dallas County | -16 | . |
| Iowa | Des Moines County | -50 | . |
| Iowa | Dickinson County | -34 | . |
| Iowa | Dubuque County | 6 | . |
| Iowa | Fayette County | -16 | . |
| Iowa | Hamilton County | -46 | . |
| Iowa | Hardin County | 58 | . |
| Iowa | Harrison County | 4 | . |
| Iowa | Henry County | -15 | . |
| Iowa | Iowa County | 13 | . |
| Iowa | Jackson County | 11 | . |
| Iowa | Jasper County | -4 | . |
| Iowa | Jefferson County | 15 | . |
| Iowa | Johnson County | -43 | . |
| Iowa | Kossuth County | -31 | . |
| Iowa | Lee County | -4 | . |
| Iowa | Linn County | -14 | . |
| Iowa | Madison County | 5 | . |
| Iowa | Mahaska County | -10 | . |
| Iowa | Marion County | 11 | . |
| Iowa | Marshall County | -8 | . |
| Iowa | Mitchell County | 33 | . |
| Iowa | Muscatine County | -61 | . |
| Iowa | Page County | -44 | . |
| Iowa | Plymouth County | -43 | . |
| Iowa | Polk County | -26 | -34 |
| Iowa | Pottawattamie County | -3 | . |
| Iowa | Poweshiek County | 7 | . |
| Iowa | Scott County | -14 | . |
| Iowa | Sioux County | 36 | . |
| Iowa | Story County | -4 | . |
| Iowa | Tama County | -53 | . |
| Iowa | Wapello County | -21 | . |
| Iowa | Warren County | 16 | . |
| Iowa | Washington County | 48 | . |
| Iowa | Webster County | -5 | . |
| Iowa | Winneshiek County | -15 | . |
| Iowa | Woodbury County | -9 | . |
| WA: Seattle (Puget Sound) | Clallam County | 5 | . |
| WA: Seattle (Puget Sound) | Grays Harbor County | -6 | . |
| WA: Seattle (Puget Sound) | Island County | 37 | . |
| WA: Seattle (Puget Sound) | Jefferson County | -20 | . |
| WA: Seattle (Puget Sound) | King County | -18 | -58 |
| WA: Seattle (Puget Sound) | Kitsap County | -23 | . |
| WA: Seattle (Puget Sound) | Mason County | 27 | . |
| WA: Seattle (Puget Sound) | Pierce County | -15 | -20 |
| WA: Seattle (Puget Sound) | San Juan County | 24 | . |
| WA: Seattle (Puget Sound) | Skagit County | 5 | . |
| WA: Seattle (Puget Sound) | Snohomish County | -16 | . |
| WA: Seattle (Puget Sound) | Thurston County | -17 | . |
| WA: Seattle (Puget Sound) | Whatcom County | -20 | . |
| Massachusetts | Barnstable County | -20 | . |
| Massachusetts | Berkshire County | -24 | . |
| Massachusetts | Bristol County | -21 | . |
| Massachusetts | Dukes County | -49 | . |
| Massachusetts | Essex County | -16 | . |
| Massachusetts | Franklin County | -9 | . |
| Massachusetts | Hampden County | -37 | 10 |
| Massachusetts | Hampshire County | -24 | . |
| Massachusetts | Middlesex County | -23 | 35 |
| Massachusetts | Norfolk County | -14 | -17 |
| Massachusetts | Plymouth County | -24 | -45 |
| Massachusetts | Suffolk County | -28 | -21 |
| Massachusetts | Worcester County | -14 | -22 |
| New Mexico | Bernalillo County | -28 | 29 |
| New Mexico | Chaves County | -50 | . |
| New Mexico | Curry County | -15 | . |
| New Mexico | Dona Ana County | -23 | . |
| New Mexico | Eddy County | -3 | . |
| New Mexico | Grant County | -39 | . |
| New Mexico | Lea County | -8 | . |
| New Mexico | Lincoln County | 35 | . |
| New Mexico | Luna County | 60 | . |
| New Mexico | Otero County | -29 | . |
| New Mexico | Sandoval County | -30 | . |
| New Mexico | San Juan County | -25 | . |
| New Mexico | Santa Fe County | -21 | . |
| New Mexico | Sierra County | 27 | . |
| New Mexico | Torrance County | -50 | . |
| New Mexico | Valencia County | -11 | . |
