# Supplementary material for: County-level racial disparities in prostate cancer–specific mortality from 2005 to 2020
Source: JNCI Cancer Spectr. 2024 Nov 4;8(6):pkae109. doi: 10.1093/jncics/pkae109 (PMC11631307; doi:10.1093/jncics/pkae109)
Supplement: pkae109_Supplementary_Data [file pkae109_supplementary_data.zip › Supplementary Table 3.docx]

**Supplementary Materials**

**Supplementary Table 3.**

**Factors associated with county-level age-adjusted prostate cancer specific mortality using generalized linear mixed regression model with negative binomial distribution in T3 (2016-2020).**

| **Variable** | **Level** | **RR (95% CI)** |
| --- | --- | --- |
| Race | Non-Hispanic White | Ref |
|  | Non-Hispanic Black | 2.09 (2.01-2.18) |
| Age group | 65+ | Ref |
|  | 50-64 | 0.1 (0.10-0.11) |
| # Primary Care Providers per 100K | <50 | Ref |
|  | >=50 | 1.02 (0.98-1.06) |
| Rural-Urban continuum | Metro areas | Ref |
|  | Urban areas | 1.02 (0.97-1.06) |
|  | Rural areas | 1.08 (0.89-1.32) |
| 4+ yr college education per 100K | <15K | Ref |
|  | 15K-30K | 0.93 (0.87-1.00) |
|  | 30K-45K | 0.91 (0.84-0.98) |
|  | >=45K | 0.89 (0.81-0.98) |
| Median household income (in dollars) | <50K | Ref |
|  | 50K-75K | 0.99 (0.95-1.03) |
|  | >=75K | 0.9 (0.84-0.96) |
| %<65 yo male without insurance | 1st quartile | Ref |
|  | 2nd quartile | 1.02 (0.98-1.06) |
|  | 3rd quartile | 1.04 (0.98-1.10) |
|  | 4th quartile | 1.01 (0.93-1.09) |
| # Urologists per 100K | <1 | Ref |
|  | 1-4 | 0.98 (0.94-1.02) |
|  | 4+ | 1.02 (0.96-1.08) |
| # Radiation Oncologists per 100K | 0 | Ref |
|  | <1 | 0.98 (0.93-1.03) |
|  | 1-2 | 0.93 (0.89-0.98) |
|  | 2-3 | 0.95 (0.90-1.01) |
|  | >3 | 0.93 (0.87-1.00) |
| # Hospital beds per 100K | 0 | Ref |
|  | <200 | 1.00 (0.91-1.09) |
|  | 200-400 | 0.98 (0.90-1.08) |
|  | 400-600 | 1.00 (0.90-1.10) |
|  | >600 | 1.02 (0.92-1.13) |
| SEER Registry | Texas | 1.00 (1.00-1.00) |
|  | San Francisco-Oakland SMSA | 1.32 (1.20-1.46) |
|  | Connecticut | 1.12 (1.04-1.20) |
|  | Hawaii | 1.19 (1.09-1.29) |
|  | Iowa | 1.30 (1.19-1.42) |
|  | New Mexico | 1.10 (0.96-1.25) |
|  | Seattle (Puget Sound) | 1.17 (1.07-1.27) |
|  | Utah | 1.28 (1.13-1.46) |
|  | Atlanta (Metropolitan) | 0.95 (0.88-1.02) |
|  | San Jose-Monterey | 1.17 (1.08-1.28) |
|  | Los Angeles | 1.38 (1.30-1.46) |
|  | Rural Georgia | 1.29 (1.07-1.55) |
|  | California excluding SF/SJM/LA | 1.30 (1.22-1.40) |
|  | Kentucky | 1.00 (0.91-1.10) |
|  | Louisiana | 0.88 (0.81-0.95) |
|  | New Jersey | 1.05 (0.98-1.12) |
|  | Greater Georgia | 0.98 (0.93-1.05) |
|  | Idaho | 1.29 (1.16-1.43) |
|  | New York | 0.98 (0.92-1.05) |
|  | Massachusetts | 1.09 (1.00-1.18) |
|  | Illinois | 1.13 (1.04-1.22) |
