# Supplementary material for: County-level racial disparities in prostate cancer–specific mortality from 2005 to 2020
Source: JNCI Cancer Spectr. 2024 Nov 4;8(6):pkae109. doi: 10.1093/jncics/pkae109 (PMC11631307; doi:10.1093/jncics/pkae109)
Supplement: pkae109_Supplementary_Data [file pkae109_supplementary_data.zip › supplementary table 2.docx]

**Supplementary Materials**

**Supplementary Table 2.**

**Factors associated with county-level age-adjusted prostate cancer specific mortality from 2005 to 2020 using generalized linear mixed regression models with negative binomial distribution with sequential nesting by SEER region (Model 1) and county within SEER region (Model 2)**

|  |  | ***Model 1*** | | ***Model 2*** | |
| --- | --- | --- | --- | --- | --- |
| **Variable** | **Level** | **RR (95% CI)** | **P** | **RR (95% CI)** | **P** |
| Race | Non-Hispanic White | Ref | <.01 | Ref | <.01 |
| . | Non-Hispanic Black | 2.37 (2.21-2.53) | . | 2.15 (2.1-2.2) | . |
| Year of death | 2005-2010 | Ref | <.01 | Ref | <.01 |
| . | 2011-2015 | 0.85 (0.83-0.86) | . | 0.85 (0.83-0.86) | . |
| . | 2016-2020 | 0.8 (0.79-0.82) | . | 0.8 (0.79-0.82) | . |
| Age group | 65+ | Ref | <.01 | Ref | <.01 |
|  | 50-64 | 0.09 (0.09-0.09) | . | 0.09 (0.09-0.09) | . |
|  | <50 | 0.09 (0.09-0.09) | . | 0.01 (0.01-0.02) | . |
| # Primary Care Providers per 100K | <50 | Ref | 0.6 | Ref | 0.6 |
| . | >=50 | 1.05 (1.02-1.08) |  | 1.05 (1.02-1.08) |  |
| # Primary Care Providers per 100K * race | >50 vs <50 among NHB | 0.94 (0.85-1.03) | <0.01 | 0.93 (0.84-1.03) | <0.01 |
|  | >50 vs <50 among NHW | 1.05 (1.02-1.08) |  | 1.05 (1.02-1.08) |  |
| Rural-Urban continuum | Metro areas | Ref | <.01 | Ref | 0.03 |
|  | Urban areas | 1.03 (1.0-1.06) | . | 1.02 (0.99-1.05) | . |
|  | Rural areas | 1.13 (1.04-1.24) | . | 1.14 (1.04-1.25) | . |
| 4+ year college education (per 100K) | <15K | Ref | <.01 | Ref | <.01 |
|  | 15K-30K | 0.92 (0.88-0.95) | . | 0.91 (0.88-0.95) | . |
|  | 30K-45K | 0.89 (0.85-0.93) | . | 0.90 (0.85-0.94) | . |
|  | >=45K | 0.89 (0.84-0.94) | . | 0.89 (0.83-0.96) | . |
| Median household income (in dollars) | <50K | Ref | <.01 | Ref | <.01 |
|  | 50K-75K | 0.99 (0.96-1.01) | . | 0.99 (0.96-1.02) | . |
|  | >75K | 0.90 (0.87-0.94) | . | 0.91 (0.86-0.96) | . |
| %<65 yo male without insurance | 1st quartile | Ref | 0.03 | Ref | 0.16 |
|  | 2nd quartile | 1.01 (0.99-1.04) | . | 1.01 (0.98-1.04) | . |
|  | 3rd quartile | 1.05 (1.01-1.06) | . | 1.04 (0.99-1.09) | . |
|  | 4th quartile | 1.01 (0.96-1.06) | . | 1.01 (0.94-1.07) | . |
| # Urologists (per 100K) | <1 | Ref | 0.2 | Ref | 0.26 |
|  | 1-4 | 0.97 (0.95-0.99) | . | 0.97 (0.94-1.01) | . |
|  | >4 | 0.99 (0.95-1.03) | . | 0.99 (0.94-1.03) | . |
| # Radiation Oncologists (per 100K) | 0 | Ref | 0.07 | Ref | 0.09 |
|  | <1 | 0.99 (0.95-1.02) | . | 0.98 (0.94-1.02) | . |
|  | 1-2 | 0.96 (0.93-0.99) | . | 0.95 (0.92-0.99) | . |
|  | 2-3 | 0.98 (0.94-1.02) | . | 0.96 (0.92-1.01) | . |
|  | >3 | 0.97 (0.93-1.01) | . | 0.97 (0.92-1.02) | . |
| # Hospital beds (per 100K) | 0 | Ref | 0.46 | Ref | 0.49 |
|  | <200 | 1.00 (0.95-1.06) | . | 0.98 (0.93-1.04) | . |
|  | 200-400 | 1.01 (0.95-1.07) | . | 0.98 (0.93-1.04) | . |
|  | 400-600 | 1.03 (0.97-1.09) | . | 1.01 (0.95-1.08) | . |
|  | >600 | 1.04 (0.97-1.11) | . | 1.01 (0.94-1.08) | . |
